# Pulmonary Artery Shear Stress and Oscillatory Shear Index are Associated with Right Ventricular Remodeling in Repaired Tetralogy of Fallot

**DOI:** 10.1007/s10439-025-03793-0

**Published:** 2025-07-03

**Authors:** Elizabeth W. Thompson, Anindro Bhattacharya, Fengling Hu, Russell T. Shinohara, Paris Perdikaris, Kevin K. Whitehead, Elizabeth Goldmuntz, Mark A. Fogel, Walter R. Witschey

**Affiliations:** 1 Advanced Cardiovascular Imaging Lab, 11-160 Perelman Center for Advanced Medicine, Perelman School of Medicine, University of Pennsylvania, Philadelphia, PA 19104 USA; 2 Department of Bioengineering, School of Engineering and Applied Sciences, University of Pennsylvania, Philadelphia, PA United States; 3 Department of Biostatistics, Epidemiology and Informatics, University of Pennsylvania, Philadelphia, PA USA; 4 Department of Mechanical Engineering and Applied Mechanics, School of Engineering and Applied Sciences, University of Pennsylvania, Philadelphia, PA USA; 5 Division of Cardiology, Department of Pediatrics, Children’s Hospital of Philadelphia, Philadelphia, PA USA; 6 Department of Radiology, Children’s Hospital of Philadelphia, Philadelphia, PA USA; 7 Department of Radiology, University of Pennsylvania, Philadelphia, PA USA

**Keywords:** Tetralogy of Fallot (ToF), Repaired tetralogy of Fallot (rToF), Computational fluid dynamics (CFD), Cardiovascular magnetic resonance (CMR), Pulmonary artery, Right ventricular remodeling

## Abstract

**Purpose:**

Right ventricular (RV) remodeling in repaired tetralogy of Fallot (rToF) is a multifactorial process that may be affected by downstream hemodynamics. We therefore sought to characterize hemodynamics in the pulmonary arteries (PAs) of rToF patients using cardiovascular magnetic resonance (CMR)-derived computational fluid dynamics (CFD) and to study these variables in association with RV measurements at follow-up.

**Methods:**

We selected patients with two CMRs who had magnetic resonance angiography (MRA) performed at baseline. The PA was segmented from the main PA (MPA) through the first bifurcation of the left PA (LPA) and right PA (RPA). Both steady and pulsatile simulations were performed. For each vessel, we calculated curvature, tortuosity, and both average (avg) and peak steady WSS (WSS_steady_), time-averaged WSS (taWSS), WSS in systole (WSS_systole_), and WSS in diastole (WSS_diastole_), as well as oscillatory shear index (OSI). We studied these variables in association with RV metrics at follow-up including: RV end-diastolic volume index (RVEDVi), RV end-systolic volume index (RVESVi), RV stroke volume index (RVSVi), and RV ejection fraction (RVEF), as well as the outcome of pulmonic valve replacement (PVR).

**Results:**

22 patients met the inclusion criteria. Several focal hemodynamic metrics in the main and branch PAs, including WSS_steady_, taWSS, WSS_systole_, WSS_diastole,_ and OSI were associated with RV measurements at follow-up, including RVEDVi, RVESVi, and RVSVi. LPA WSS_steady,avg_, RPA WSS_steady,peak_, whole vessel OSI_avg_, and MPA OSI_avg_ were associated with likelihood of PVR.

**Conclusion:**

CFD-derived hemodynamic variables in the PAs of rToF patients are associated with both PVR and RV remodeling.

**Supplementary Information:**

The online version of this article contains supplementary material available 10.1007/s10439-025-03793-0.

## Introduction

Tetralogy of Fallot (ToF) is a cyanotic congenital heart disease characterized by four defects, one of which is right ventricular outflow tract (RVOT) obstruction leading to RV hypertrophy. Repairing ToF resolves the RVOT obstruction and RV hypertrophy; however, these patients often have concomitant branch pulmonary artery (PA) stenosis [1]. Additionally, RVOT stenosis can reoccur after initial repair [2]. These changes increase the right heart afterload, contributing to worsening RV structural and functional parameters. The manifestations of these can include pulmonary regurgitation (PR), ventricular dilation, and eventual right heart failure [3]. Additionally, some patients will receive pulmonary valve replacement (PVR) to improve symptoms, lessen PR, and decrease RV dilation [4].

Many patients with repaired ToF (rToF) receive magnetic resonance angiography (MRA) imaging to delineate the anatomy of the thoracic vessels as part of a cardiovascular magnetic resonance (CMR) scan, as ToF is associated with other vascular anomalies such as right sided aortic arch and systemic venous anomalies [5]. The high spatial resolution of these scans enables detailed visualization of the pulmonary vasculature, which can then be used to create geometric models for computational fluid dynamics (CFD) simulations [6]. CFD is an in-silico collection of methods that allows quantification of flow parameters throughout a geometry by solving the Navier-Stokes equations, enabling the measurement of detailed hemodynamic variables [7].

Previous CFD work in rToF has shown an association of the geometric index centerline torsion with the CFD-derived variable of energy efficiency [8]. Another study showed that larger pre-operative PA bifurcation angle was associated with risk of reoperation in rToF [9]. One important CFD-derived metric is wall shear stress (WSS), the tangential force of blood along the vessel wall. WSS has been shown to increase nitric oxide signaling in the endothelium, leading to vasodilation [10]. Non-physiologic WSS has also been shown to impact endothelial cell morphology and gene expression, and may lead to the development of intimal hyperplasia [11]. Another local flow metric is oscillatory shear index (OSI), which quantifies how much the direction of WSS changes throughout the cardiac cycle. OSI may disturb signaling pathways associated with homeostasis, leading to the activation of proinflammatory signaling in the endothelium [11]. Additionally, OSI is likely elevated in rToF compared to normal PAs due to the high amount of PR, which reverses the direction PA flow during the cardiac cycle. Together, these alterations lead to changes in afterload, which can alter the geometry and systolic motion of the RV [12]. These prior findings indicate that local hemodynamic factors such as WSS and OSI may play a role in RV remodeling in rToF.

While WSS has been well-studied in pulmonary arterial hypertension, few other pulmonary vascular diseases have been investigated using CFD. Additionally, the CFD studies in rToF to-date have only characterized hemodynamics at a single timepoint [8, 13]. Given the frequency of altered pulmonary vasculature in rToF, combined with irregular flow due to PR, these patients’ hearts are subjected to highly abnormal hemodynamic stressors over many years. We therefore sought to investigate the relationship between RV remodeling and CFD-derived PA hemodynamic measures, including WSS and OSI, hypothesizing that these metrics would be associated with RV remodeling and PVR. We tested this hypothesis by performing CFD on PAs from baseline CMR scans and analyzing the resulting hemodynamic measures in association with RV structural and functional measurements at a second scan, as well as PVR-free survival after initial scan.

## Materials and Methods

### Patients

The study population comes from a longitudinal cohort of patients with rToF who received CMR imaging between January 2000 and December 2020 (the Single Center Outcomes Study in tetralogy of Fallot [SCOUT-TOF]) at the Children’s Hospital of Philadelphia (CHOP; Philadelphia, Pennsylvania, United States). Deidentified scans were retrospectively collected for consecutive rToF patients. Inclusion criteria were: (1) cardiac MRA imaging performed in systole, and (2) presence of a second CMR to assess structural and functional changes over time. Patients were excluded if they did not meet these criteria or if the image quality was inadequate for PA segmentation. We further stratified patients by whether they received a pulmonic valve replacement (PVR) by the end of the study period. Time to PVR was calculated as the number of days between the patient’s first CMR and PVR or the date of last contact, as appropriate. Patients who received a PVR between Scan 1 and Scan 2 were excluded from studies associating baseline parameters with remodeling, as PVR was expected alter the trajectory of RV remodeling. The study protocol was approved by the Institutional Review Board of CHOP, and has been carried out in accordance with the Declaration of Helsinki. Informed consent was waived due to the retrospective nature of the study.

### Image Acquisition

All CMRs were acquired in a 1.5T Sonata, Avanto, or Avanto FIT Whole Body Magnetic Resonance System (Siemens Healthcare, Erlangen, Germany). MRA was acquired using a cardiac and respiratory-navigated fast low angle shot sequence in systole using either gadopentetate dimeglumine 0.4 cc/kg or gadobutrol 0.2 cc/kg contrast. Images were acquired as a sagittal stack with a slice thickness ranging from 1.1 to 1.3 mm and an in-plane resolution ranging from 0.34 to 1.76 mm^2^ per pixel. Other sequence parameters were: repetition time (TR) 251–357 ms, echo time (TE) 1.48–1.79 ms, bandwidth 490–501 Hz, flip angle 18°, and field of view (FOV) 230–420 × 150–270 mm.

Through-plane, retrospectively gated, phase contrast magnetic resonance imaging (PC-MRI) was used to assess flow in the PAs. Velocity encoding was generally 250 cm/s (ranging from 150 to 350 cm/s) and was adjusted at the performing radiologist’s discretion. Other sequence parameters were: slice thickness 4–5 mm, TR 27–59 ms, TE 2.5–3.4 ms, bandwidth 500–705 Hz, flip angle 25°, FOV 165–350 × 169–360, and in-plane resolution of 1.03–2.44 mm^2^ per pixel.

To assess cardiac function, short-axis balanced steady-state free precession images were acquired in 8-12 contiguous slices from cardiac base to apex, with an in-plane resolution of 0.6–2.5 mm and a flip angle of 58 to 90°. 20–30 cardiac phases were captured, depending on the patient’s heart rate, and image acquisition was cardiac-triggered. Parallel imaging was performed with an acceleration factor of 2. Other sequence parameters were: slice thickness 6–10 mm, TR 18–53 ms, TE 1.3–1.8 ms, bandwidth 930–1185 Hz, temporal resolution 22–55 ms, and FOV 200–400 × 150–300 mm. Short-axis cine images were analyzed to derive RV structural and functional parameters.

### Pulmonary Artery Modeling

PA models were generated starting proximally at the main PA (MPA) extending to the first bifurcation in the left PA (LPA) and right PA (RPA) using SimVascular (version 2023.03.27, RRID:SCR_002686) [14]. To establish the flow field at the inlet and outlets, extensions of length five times the diameter of the respective inlet or outlet were added to each face using the Vascular Modeling Toolkit (VMTK, version 1.4.0, vmtk.org, RRID:SCR_001893) [15]. The solid models with extensions were re-imported into SimVascular and meshed using isotropic tetrahedra to produce meshes of approximately eight million elements. We performed mesh independence testing on three patients to confirm stability of WSS measurements at this mesh density (see Supplemental Figure 1).

**Fig. 1:**
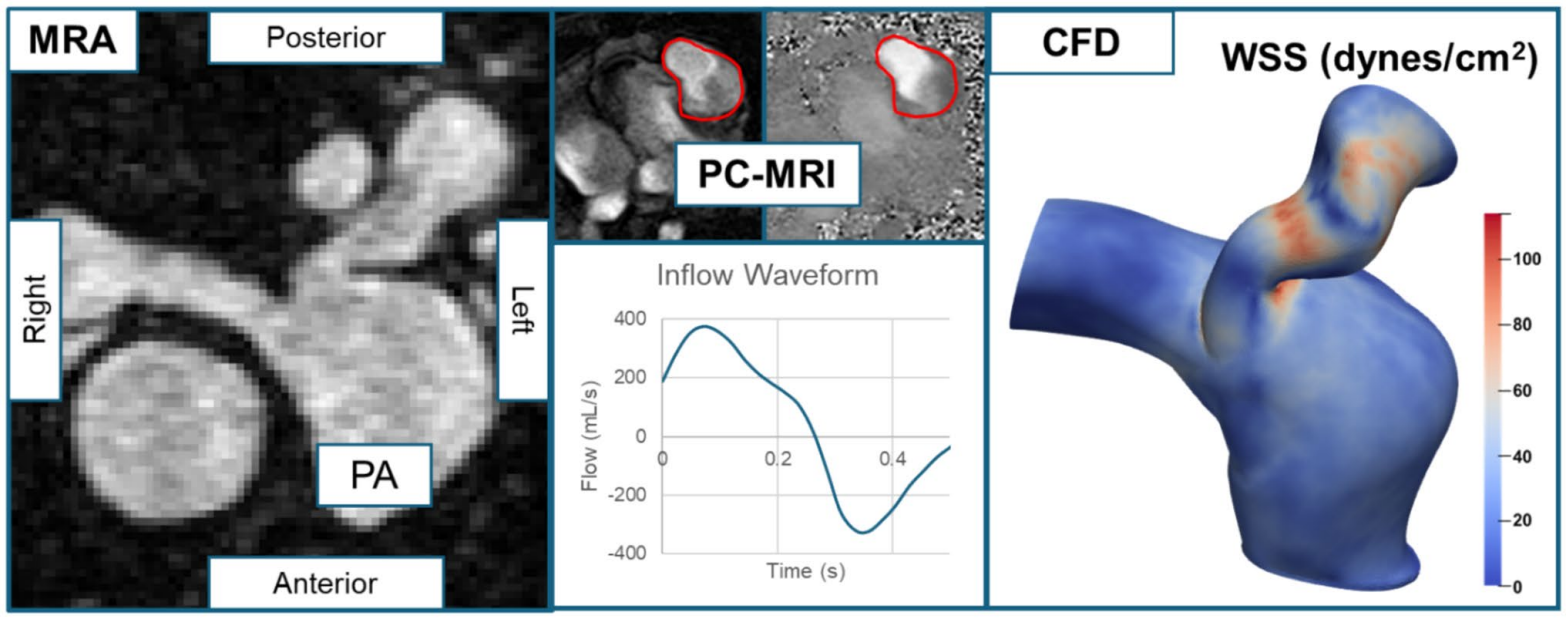
Pulmonary artery modeling and computational fluid dynamics pipeline. The pulmonary artery (PA) was segmented from magnetic resonance angiography (MRA) acquired in systole. Phase-contrast magnetic resonance imaging (PC-MRI) was used to extract the flow waveform at the inlet, which was applied as a periodic boundary condition. Computational fluid dynamics (CFD) simulation was performed, and hemodynamic variables of interest, such as wall shear stress (WSS; shown here from steady simulations) were outputted and studied in association with pulmonic valve replacement and right ventricular remodeling

### Boundary Conditions and CFD Simulations

We performed both steady and pulsatile inlet simulations. For steady simulations, the inflow was calculated as (stroke volume × heart rate). For pulsatile simulations, physicians calculated the pulsatile flow waveform at the MPA using cvi42 (Calgary, Alberta, Canada) as part of routine reporting on CMR. First, the physician segmented the MPA in the MPA PC-MRI magnitude images. Cvi42 then automatically integrated the velocity values from the phase image across the segmentation for each time point, generating a flow waveform. This waveform was then mapped onto a parabolic velocity profile at the inlet in SimVascular, using ten Fourier nodes to characterize the periodic flow in the frequency domain [14].

The remaining boundary conditions were kept the same in both steady and pulsatile simulations. A resistance boundary condition of 2 indexed Wood units [16] was used at both the LPA and RPA outlets to achieve a total resistance of 1 indexed Wood unit for the lungs.

The walls were assumed to be rigid, and a no-slip condition was applied. Blood was assumed to be a Newtonian fluid with density = 1.06 g/cm^3^ and viscosity = 0.04 g/cm·s (the default values set by SimVascular) [14, 17].

For steady simulations, we simulated 2.5 seconds of flow at a time step of 0.0001 seconds. While this is computationally intense for steady-state simulations, this allowed us to directly compare numerical stability and convergence behavior across all cases. Supplemental Figure 2 plots the log non-linear residual over iterations, and Supplemental Table 1 shows the change in non-linear residual over the last 100 iterations of the simulation. For pulsatile simulations, five cardiac cycles were simulated based on the patient’s heart rate and a time step of 0.0001 seconds. This time step size was chosen to ensure convergence of results based on the Courant–Friedrichs–Lewy condition [18] and was kept consistent between steady and pulsatile simulations. Convergence in the pulsatile simulations was determined by examining the periodicity of the non-linear residual. At both systole and diastole, the log residual did not change more than 7% over the last two simulated cycles (Supplemental Figure 2). The periodic nature is also visualized in Supplemental Figure 3. All simulations were run on the SimVascular Gateway (gateway.simvascular.org) [19].

### CFD Post-processing

For each model, the centerline geometry for both MPA to LPA and MPA to RPA was generated using VMTK. Each centerline was then resampled at increments of 0.1 cm and processed using VMTK to measure curvature (the inverse of the radius of the local osculating circle) at each point (Equation 1) and average curvature for the centerline was reported [20]. Tortuosity was calculated as in Equation 2 [20].

1 $$\kappa \left(s\right)= \frac{\left|{c}{\prime}\left(s\right)\times c{\prime}{\prime}(s)\right|}{{\left|{c}{\prime}\left(s\right)\right|}^{3}}$$

Equation 1: Curvature calculation. Where κ is the curvature, c is the centerline, and s is the point along the centerline [20].

2 $${\rm X}= \frac{L}{D}-1$$

Equation 2: Tortuosity calculation. Where Χ is the tortuosity, L is the length of the centerline, and D is the Euclidean distance between the endpoints of the centerline [20].

WSS was calculated in SimVascular for each point on the wall of the mesh (Equation 3).

3 $$WSS= \mu \frac{du}{dy}{|}_{y=0}$$

Equation 3: Wall shear stress calculation. Where WSS is wall shear stress, μ is the viscosity of blood, and du/dy is the derivative of velocity (u) at the vessel wall (y = 0) [21].

For steady simulations, results were outputted at the last time step. For pulsatile simulations, results were outputted for twenty evenly-spaced timepoints across the last simulated cardiac cycle. Vessel extensions were trimmed in Paraview, and VMTK was used to automatically divide vessel models into segments corresponding to the MPA, LPA, and RPA. For steady flow conditions, average (WSS_steady,avg_) and peak WSS (WSS_steady,peak_) were calculated for each vessel segment as well as the whole vessel. From the pulsatile flow conditions, we used the inflow data to determine WSS at the systolic (maximum) and diastolic (minimum) flow timepoints, then calculated average (WSS_systole,avg_ and WSS_diastole,avg_) and peak WSS (WSS_systole,peak_ and WSS_diastole,peak_) at these timepoints. We also calculated time-averaged WSS (taWSS; pulsatile WSS averaged across the cardiac cycle), again reporting average (taWSS_avg_) and peak (taWSS_peak_) values.

We also determined oscillatory shear index (OSI; **Equation **4) from the pulsatile results, and calculated average OSI (OSI_avg_) across the whole vessel, MPA, LPA, and RPA.

4 $$OSI({\varvec{x}})=\frac{1}{2}\left(1-\frac{\left|{\int }_{0}^{T}WSS({\varvec{x}},t)dt\right|}{{\int }_{0}^{T}\left|WSS({\varvec{x}},t)\right|dt}\right)$$

Equation 4: Oscillatory shear index calculation. Where OSI is the oscillatory shear index, WSS is the wall shear stress, x is the point in 3D space, t is the time during the cardiac cycle, and T is the length of the cardiac cycle [11].

An OSI of 0 indicates that the WSS is in the same direction throughout the cardiac cycle, while an OSI of 0.5 (the highest possible value of OSI) indicates that the WSS changes direction considerably during the cardiac cycle.

The pipeline from imaging to CFD simulation is shown in Figure 1.

### Statistics

Categorical variables are expressed as N (%); continuous variables are expressed as median [Q1, Q3]. Normality testing was performed using the Shapiro-Wilk test. Categorical variables were compared using chi-square testing, and continuous variables were compared using unpaired student’s t-test, Wilcoxon signed rank test, or Kruskal-Wallis test as appropriate based on the variables and distributions of the data. For intra-patient comparisons (e.g. MPA vs. LPA WSS in the same patient), paired student’s t-test or paired Wilcoxon tests were used as appropriate based on the data distribution.

#### Primary Analysis

In patients with no PVR between scans, we performed multivariable linear regression of baseline geometric and CFD-derived measures in association with RV measurements at a second scan. These metrics included: RV end-diastolic volume index (RVEDVi), RV end-systolic volume index (RVESVi), RV stroke volume index (RVSVi), and RV ejection fraction (RVEF). Each model was adjusted for patient sex, age at CMR1, and age at CMR2. P-values were adjusted for multiple comparisons using the Benjamini–Hochberg method.

#### Secondary Analysis

Multivariable Cox proportional hazards models were used to characterize the relationships between baseline geometric and CFD-derived measures and time to PVR. Covariables were chosen *a priori* as patient age at CMR and patient sex. The proportional hazards assumption was tested by investigating the Schoenfeld residuals, and was found to be valid for each variable. For variables found to be significant in the multivariable analysis, we performed receiver operating characteristic (ROC) analysis to determine an optimal cutoff for the respective variable to predict PVR over the median time to PVR in the cohort. The optimal cutoff was chosen as the point at which the sum of sensitivity and specificity was maximized. Patients were then classified as “above” or “below” this cutoff for each variable, and Kaplan Meier analysis was used to visualize the differences in PVR-free survival between these groups.

P values less than 0.05 were considered statistically significant. All statistical analysis was performed using R 4.4.0 (R Foundation for Statistical Computing, Vienna, Austria, RRID:SCR_000432).

## Results

### Baseline Characteristics of the Cohort

**Fig. 2:**
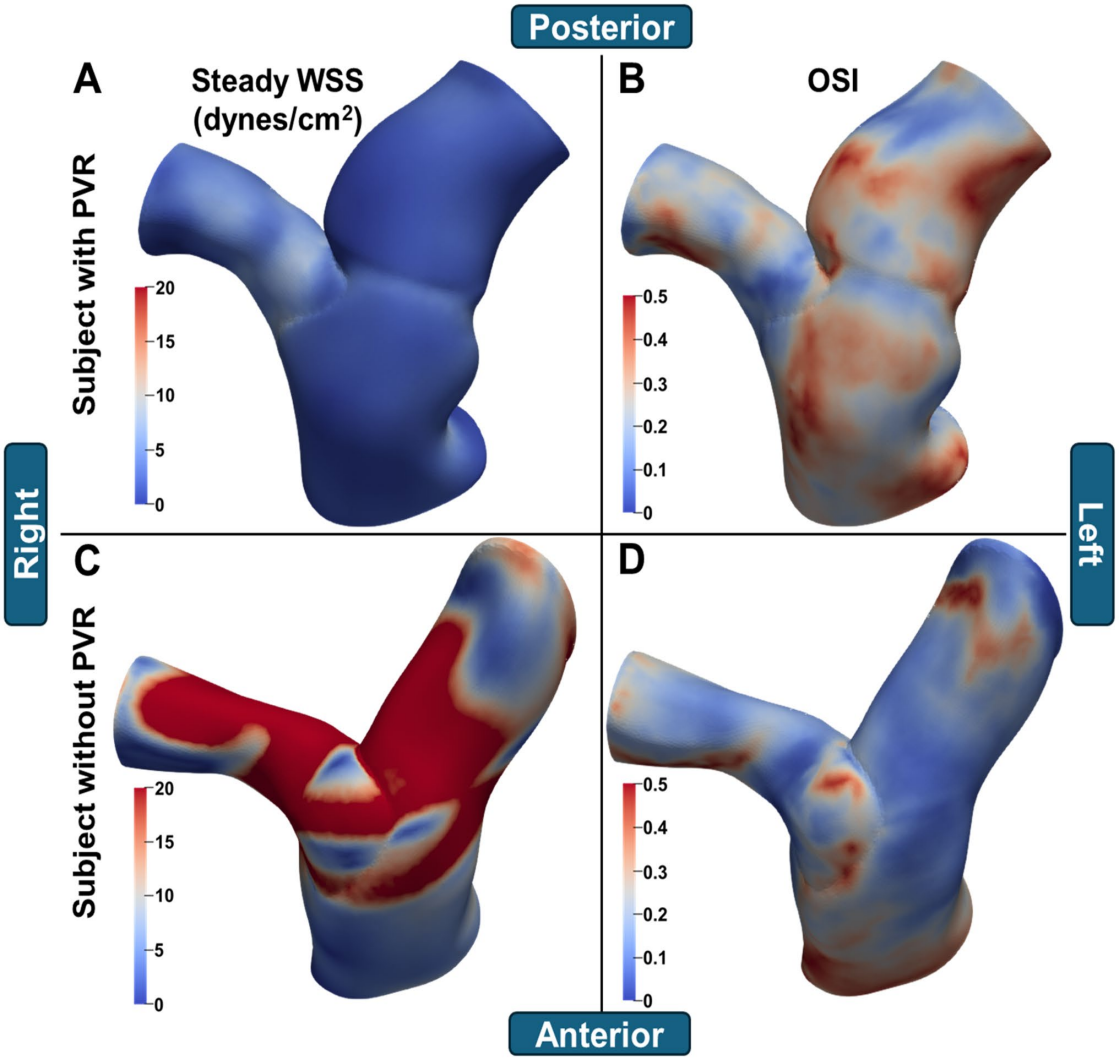
Sample steady WSS and OSI maps from rToF patients. Color maps are shown for two subjects: one who received PVR (**A** and **B**), and one who did not (**C** and **D**). Steady WSS is shown in the left column, while OSI is shown on the right. The first subject had lower WSS (**A**) and higher OSI (**B**), while the second subject had higher WSS (**C**) and lower OSI (**D**). *OSI* oscillatory shear index*, PVR* pulmonic valve replacement*, WSS* wall shear stress

Table 1 contains the baseline characteristics of the study cohort. Twenty-two patients met the inclusion criteria. Patients received their first CMRs at a median of 12 years of age (Q1-Q3 7.3-15.6 years), and 13 (59%) were female. There was a median of 3.5 (Q1-Q3 2.9-4.5) years between CMR scans. The majority (77%) identified as Caucasian, and most (86.4%) received a transannular patch repair. Five patients received a PVR before their second CMR, and one patient received a PVR after their second CMR. WSS and OSI results for 2 cases are shown in Figure 2. Baseline geometry and hemodynamic measurements for the whole cohort are shown in Table 2.

**Table 1:** Baseline characteristics of the study cohort

	Overall (N = 22)
Sex	
Female	13 (59.1%)
Male	9 (40.9%)
Age at initial repair (years)	0.39 [0.25, 1.09]
Age at CMR (years)	12.0 [7.3, 15.6]
Time between scans (years)	3.54 [2.89, 4.48]
Ethnicity	
African-American	2 (9.1%)
Caucasian	17 (77.3%)
Hispanic	2 (9.1%)
Other	1 (4.5%)
Type of initial repair	
Conduit	1 (4.5%)
No Transannular patch	1 (4.5%)
Transannular patch	19 (86.4%)
Unknown	1 (4.5%)
RVEDVi (mL/m^2^)	145 [121, 163]
RVESVi (mL/m^2^)	64.6 [51.7, 79.5]
RVSVi (mL/m^2^)	70.8 [64.7, 91.1]
RVEF (%)	56.6 [48.9, 60.0]
Pulmonary RF	0.400 [0.323, 0.475]

The study population consisted of 22 repaired tetralogy of Fallot subjects who had (1) a post-repair baseline magnetic resonance angiography scan performed in systole, and (2) a follow-up CMR scan with cardiac function imaging. Values are shown as n (%) or median [Q1–Q3]. *CMR* cardiovascular magnetic resonance imaging, *EDV* end-diastolic volume*, EF* ejection fraction*, ESV* end-systolic volume, *i index*, *RF* regurgitant fraction, *RV* right ventricle.

**Table 2:** Geometry and hemodynamics for the overall cohort

Variable	Whole vessel	MPA	LPA	RPA
Curvature (cm^− 1^)	–	–	0.968 [0.781, 1.16]	0.821 [0.711, 0.897]
Tortuosity	–	–	0.222 [0.135, 0.343]	0.193 [0.155, 0.225]
WSS_steady,avg_ (dynes/cm^2^)	6.16 [5.41, 7.59]	4.08 [2.60, 4.83]	11.3 [7.76, 14.6]	6.77 [5.25, 9.08]
WSS_steady,peak_ (dynes/cm^2^)	41.8 [25.9, 49.0]	25.8 [19.9, 40.6]	38.3 [25.8, 48.5]	24.1 [16.2, 34.3]
taWSS_avg_ (dynes/cm^2^)	17.6 [14.1, 21.2]	12.4 [9.89, 15.6]	26.1 [20.3, 33.4]	17.8 [13.9, 24.5]
taWSS_peak_ (dynes/cm^2^)	80.5 [59.0, 109]	59.5 [42.8, 82.4]	76.0 [59.0, 102]	52.9 [41.8, 79.4]
WSS_systole,avg_ (dynes/cm^2^)	50.9 [40.1, 59.9]	34.4 [24.7, 39.5]	69.6 [57.9, 103]	54.6 [42.5, 72.3]
WSS_systole,peak_ (dynes/cm^2^)	209 [162, 295]	161 [94.5, 201]	194 [160, 288]	150 [120, 198]
WSS_diastole,avg_ (dynes/cm^2^)	23.8 [14.0, 31.4]	21.6 [14.6, 28.9]	24.3 [13.9, 39.2]	23.1 [12.0, 28.7]
WSS_diastole,peak_ (dynes/cm^2^)	104 [69.7, 144]	93.0 [69.6, 125]	89.5 [51.0, 138]	69.4 [53.7, 102]
OSI_avg_	0.214 [0.200, 0.226]	0.243 [0.229, 0.259]	0.167 [0.152, 0.196]	0.189 [0.167, 0.199]

Geometric and CFD-derived hemodynamic variables characterizing the overall cohort are reported as median [Q1, Q3]

*Avg* average, *LPA* left pulmonary artery, *MPA* main pulmonary artery, *OSI* oscillatory shear index, *RPA* right pulmonary artery, *taWSS* time-averaged wall shear stress, *WSS* wall shear stress.

#### Comparison of Geometric and Hemodynamic Parameters in the MPA, RPA, and LPA

**Fig. 3:**
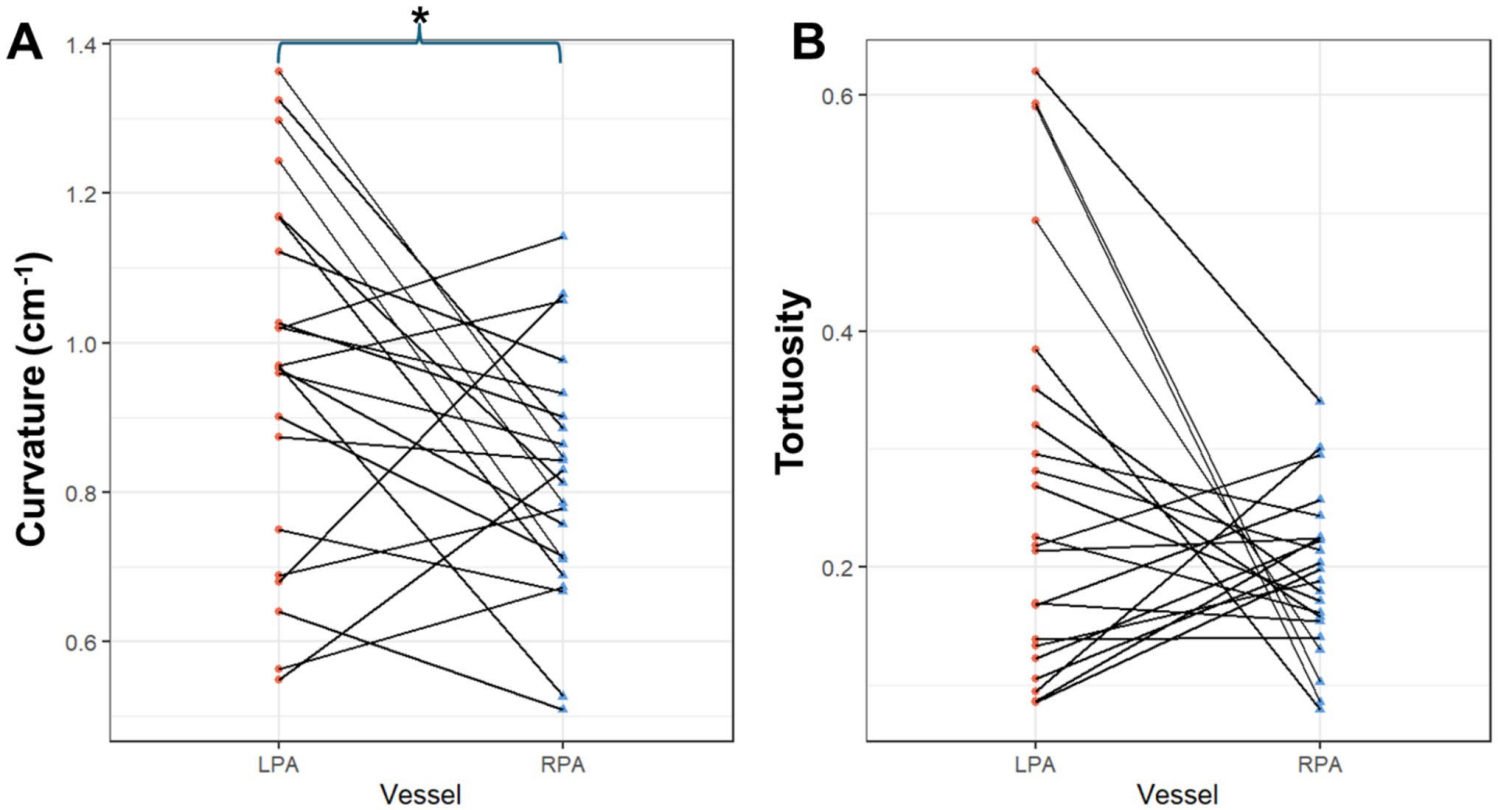
Centerline geometry comparisons between the left and right pulmonary arteries in patients with rToF. Centerlines were extracted from the segmented pulmonary artery (PA) models using the Vascular Modeling Toolkit. The LPA path consisted of the beginning of the MPA through the end of the LPA, and the RPA path consisted of the beginning of the MPA through the end of the RPA. Curvature (**A**) differed significantly between the LPA (red) and RPA (blue), while tortuosity (**B**) did not. *LPA* left pulmonary artery, *MPA* main pulmonary artery, *RPA* right pulmonary artery, *rToF* repaired tetralogy of Fallot. *p < 0.05

LPA curvature was significantly higher than RPA curvature (0.97 vs. 0.82 cm^−1^, p = 0.015, Figure 3a), while tortuosity was not different between these vessel segments (p = 0.26, Figure 3b).

**Fig. 4:**
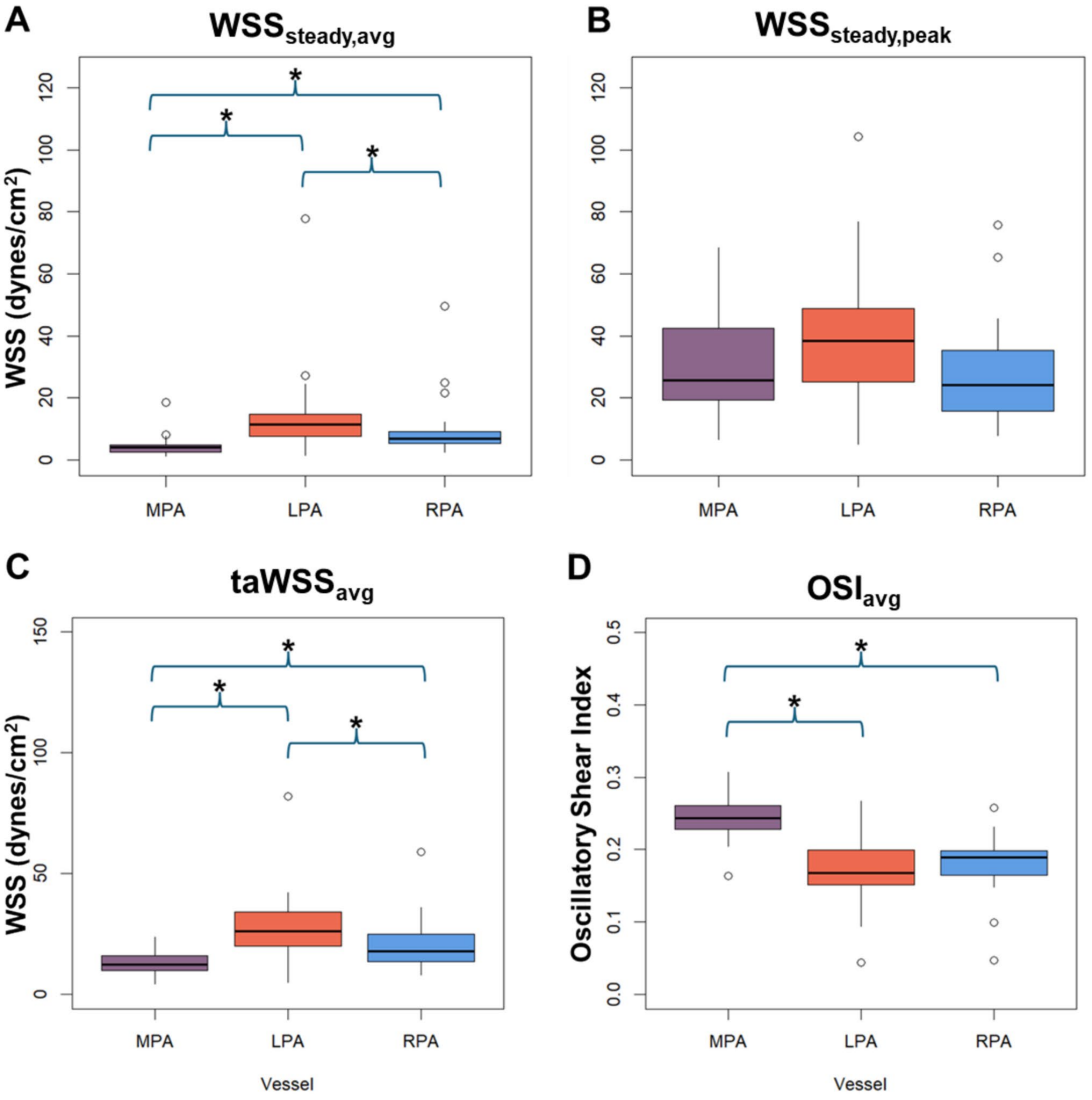
Steady and Pulsatile CFD-derived measurement comparisons between the main, left, and right pulmonary arteries in patients with rToF. Vessel-specific average (avg) and peak wall shear stress (WSS) and oscillatory shear index (OSI) were extracted from steady and pulsatile CFD simulations performed on pulmonary artery models from patients with rToF. For WSS_steady,avg_ and taWSS_avg_, the LPA (red) had greater WSS than both the RPA (blue) and MPA (purple), and RPA WSS was greater than MPA WSS (**A** and **C**). WSS_steady,peak_ did not differ significantly between vessels (**B**). Additionally, the MPA had higher OSI than both the LPA and RPA (**D**). *CFD* computational fluid dynamics, *LPA* left pulmonary artery, *MPA* main pulmonary artery, *RPA* right pulmonary artery, *rToF* repaired tetralogy of Fallot. *p < 0.05

From the steady simulations, we compared WSS_steady,avg_ between the MPA, LPA, and RPA for each patient and found significant differences (Figure 4a; p < 0.010). Post-hoc pairwise comparisons showed statistically significant differences between all vessels (p < 0.010 for all), with the LPA having the highest WSS_steady,avg_ (11.3 dynes/cm^2^), followed by the RPA (6.77 dynes/cm^2^), then the MPA (4.08 dynes/cm^2^). In contrast, WSS_steady,peak_ did not vary between vessel segments (Figure 4b).

From the pulsatile results, we compared taWSS, WSS_systole_, WSS_diastole_, and OSI_avg_ between vessel segments. taWSS_avg_ was different between all vessel segments (p < 0.010 for Kruskal-Wallis) and was highest in the LPA (26.1 dynes/cm^2^), followed by the RPA (17.8 dynes/cm^2^), and the MPA (12.4 dynes/cm^2^; Figure 4c), while taWSS_peak_ did not differ between these groups (Supplemental Figure 4). Likewise, WSS_systole,avg_ was different across vessels (p < 0.010); again highest in the LPA (69.6 dynes/cm^2^), then RPA (54.6 dynes/cm^2^) and MPA (34.4 dynes/cm^2^), and WSS_systole,peak_ was not different between segments (Supplemental Figure 5). Interestingly, neither WSS_diastole,avg_ nor WSS_diastole,peak_ differed between vessel segments (Supplemental Figure 6), while OSI_avg_ varied significantly (p < 0.010; Figure 4d); MPA OSI_avg_ was greater than both LPA and RPA OSI_avg_, but LPA and RPA OSI_avg_ were not significantly different.

**Fig. 5:**
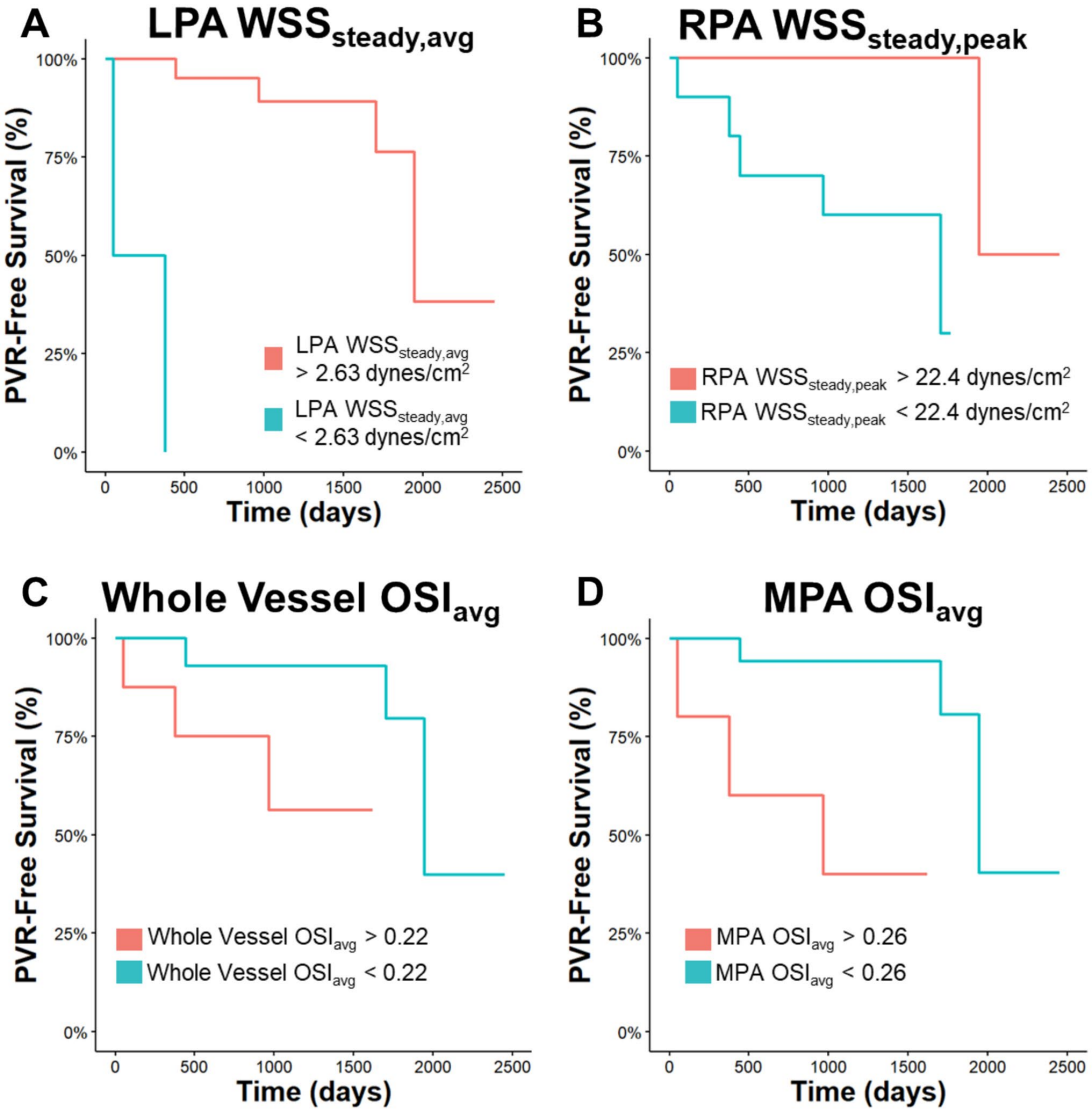
Kaplan Meier curves for CFD-derived metrics to predict pulmonary valve replacement. We used multivariable Cox proportional hazards analyses adjusting for patient sex and age at CMR to identify CFD-derived variables that were predictive of PVR. Receiver operating characteristic analysis was then used to determine optimal cutoffs for each significant variable to predict PVR, and Kaplan Meier curves were constructed from these cutoffs. Variables included: LPA WSS_steady,avg_ (**A**, cutoff 2.63 dynes/cm^2^), RPA WSS_steady,peak_ (**B**, cutoff 22.4 dynes/cm^2^), whole vessel OSI_avg_ (**C**, cutoff 0.22), and MPA OSI_avg_ (**D**, cutoff 0.26). *CFD* computational fluid dynamics, *CMR* cardiovascular magnetic resonance, *LPA* left pulmonary artery, *MPA* main pulmonary artery, *OSI* oscillatory shear index, *PVR* pulmonary valve replacement, *RPA* right pulmonary artery, *WSS* wall shear stress

#### Primary Analysis: Geometric and CFD-Derived Variables in Association with RV Remodeling Metrics

Seventeen patients had no PVR between their two scans and were studied in the primary analysis. In multivariable linear regression models adjusting for age at baseline CMR, age at follow-up CMR, and patient sex (and after adjusting for multiple comparisons), we found that RVEDVi at second scan was associated with several focal CFD metrics including: whole vessel, MPA, LPA, and RPA WSS_steady_, whole vessel and RPA taWSS, RPA WSS_systole_, whole vessel, MPA, LPA, and RPA WSS_diastole_, and whole vessel and RPA OSI (Table 3). RVESVi at second scan was associated with whole vessel, MPA, and RPA WSS_steady,avg_, and RPA WSS_steady,peak_ and taWSS_peak_ (Table 4)_._ RVSVi at second scan was associated with whole vessel, MPA, LPA, and RPA WSS_steady_, RPA taWSS_avg_, whole vessel and RPA taWSS_peak_, RPA WSS_systole_, whole vessel, MPA, LPA, and RPA WSS_diastole_, and whole vessel and RPA OSI (Table 5). No variables were associated with RVEF at second scan (Table 6).

**Table 3:** Multivariable linear regression results for RVEDVi at Scan 2

Variable	Whole vessel		MPA		LPA		RPA	
	Effect Estimate	Unadjusted p-value	Effect Estimate	Unadjusted p-value	Effect Estimate	Unadjusted p-value	Effect Estimate	Unadjusted p-value
Curvature* (cm^−1^)	–	–	–	–	43.7	0.409	46.9	0.550
Tortuosity*	–	–	–	–	45.8	0.537	− 90.9	0.603
WSS_steady,avg_ (dynes/cm^2^)	− 4.74	** < 0.001**	− 9.64	** < 0.001**	− 1.71	**0.011**	− 3.36	** < 0.001**
WSS_steady,peak_ (dynes/cm^2^)	− 0.473	**0.003**	− 0.758	**0.011**	− 0.434	**0.007**	− 0.719	** < 0.001**
taWSS_avg_ (dynes/cm^2^)	− 4.09	**0.025**	− 3.28	0.321	− 1.4	0.082	− 3.03	**0.003**
taWSS_peak_ (dynes/cm^2^)	− 0.452	**0.014**	− 0.491	0.110	− 0.39	0.033	− 0.735	**0.001**
WSS_systole,avg_ (dynes/cm^2^)	− 1.42	0.043	− 2.03	0.058	− 0.37	0.157	− 1.08	**0.023**
WSS_systole,peak_ (dynes/cm^2^)	− 0.136	0.047	− 0.197	0.119	− 0.118	0.083	− 0.266	**0.003**
WSS_diastole,avg_ (dynes/cm^2^)	3.18	**0.002**	3.09	**0.005**	1.75	**0.009**	2.8	**0.007**
WSS_diastole,peak_ (dynes/cm^2^)	0.563	**0.003**	0.569	**0.003**	0.545	**0.004**	0.734	**0.013**
OSI_avg_	1090	**0.011**	543	0.245	402	0.146	571	**0.014**

Multivariable linear regression adjusting for patient sex, age at scan 1, and age at scan 2 was performed between each geometric and hemodynamic variable (with different vessel locations indicated by column) and the continuous outcome of RVEDVi at Scan 2. Subjects with PVR between scans were excluded from this analysis. Effect estimates are interpreted as change in RVEDVi (mL/m^2^) per unit increase in the baseline variable. Unadjusted p-values are provided. p-values that remained significant after Benjamini–Hochberg adjustment are bolded

*CI* confidence interval, *CMR* cardiovascular magnetic resonance, *LPA* left pulmonary artery, *MPA* main pulmonary artery, *OSI* oscillatory shear index, *OR* odds ratio, *RPA* right pulmonary artery, *taWSS* time-averaged wall shear stress, *WSS* wall shear stress.

**Table 4:** Multivariable linear regression results for RVESVi at Scan 2

Variable	Whole Vessel		MPA		LPA		RPA	
	Effect estimate	Unadjusted p-value	Effect estimate	Unadjusted p-value	Effect estimate	Unadjusted p-value	Effect estimate	Unadjusted p-value
Curvature* (cm^−1^)	–	–	–	–	28.2	0.343	15.8	0.722
Tortuosity*	–	–	–	–	48.5	0.236	− 59.1	0.548
WSS_steady,avg_ (dynes/cm^2^)	− 2.38	**0.005**	− 4.99	**0.002**	− 0.887	0.023	− 1.63	**0.005**
WSS_steady,peak_ (dynes/cm^2^)	− 0.237	0.012	− 0.414	0.016	− 0.221	0.019	− 0.368	**0.002**
taWSS_avg_ (dynes/cm^2^)	− 2.17	0.038	− 2.08	0.262	− 0.762	0.095	− 1.48	0.014
taWSS_peak_ (dynes/cm^2^)	− 0.233	0.029	− 0.304	0.075	− 0.202	0.055	− 0.382	**0.005**
WSS_systole,avg_ (dynes/cm^2^)	− 0.79	0.047	− 1.24	0.037	− 0.214	0.145	− 0.539	0.050
WSS_systole,peak_ (dynes/cm^2^)	− 0.0704	0.072	− 0.123	0.081	− 0.0629	0.105	− 0.138	0.009
WSS_diastole,avg_ (dynes/cm^2^)	1.52	0.013	1.49	0.022	0.825	0.037	1.25	0.044
WSS_diastole,peak_ (dynes/cm^2^)	0.266	0.019	0.27	0.018	0.262	0.022	0.311	0.081
OSI_avg_	515	0.041	275	0.299	158	0.322	263	0.057

Multivariable linear regression adjusting for patient sex, age at scan 1, and age at scan 2 was performed between each geometric and hemodynamic variable (with different vessel locations indicated by column) and the continuous outcome of RVESVi at Scan 2. Subjects with PVR between scans were excluded from this analysis. Effect estimates are interpreted as change in RVESVi (mL/m^2^) per unit increase in the baseline variable. Unadjusted p-values are provided. p-values that remained significant after Benjamini–Hochberg adjustment are bolded

*CI* confidence interval, *CMR* cardiovascular magnetic resonance, *LPA* left pulmonary artery, *MPA* main pulmonary artery, *OSI* oscillatory shear index, *OR* odds ratio, *RPA* right pulmonary artery, *taWSS* time-averaged wall shear stress, *WSS* wall shear stress.

**Table 5:** Multivariable linear regression results for RVSVi at Scan 2

Variable	Whole Vessel		MPA		LPA		RPA	
	Effect estimate	Unadjusted p-value	Effect estimate	Unadjusted p-value	Effect estimate	Unadjusted p-value	Effect estimate	Unadjusted p-value
Curvature* (cm^−1^)	–	–	–	–	15.6	0.554	31	0.418
Tortuosity*	–	–	–	–	− 2.71	0.941	− 31.8	0.713
WSS_steady,avg_ (dynes/cm^2^)	− 2.35	** < 0.001**	− 4.64	** < 0.001**	− 0.827	**0.013**	− 1.73	** < 0.001**
WSS_steady,peak_ (dynes/cm^2^)	− 0.236	**0.002**	− 0.345	**0.023**	− 0.213	**0.008**	− 0.351	** < 0.001**
taWSS_avg_ (dynes/cm^2^)	− 1.93	0.034	− 1.2	0.467	− 0.639	0.111	− 1.54	**0.002**
taWSS_peak_ (dynes/cm^2^)	− 0.22	**0.016**	− 0.186	0.230	− 0.189	0.037	− 0.353	**0.002**
WSS_systole,avg_ (dynes/cm^2^)	− 0.63	0.074	− 0.789	0.147	− 0.156	0.231	− 0.538	**0.021**
WSS_systole,peak_ (dynes/cm^2^)	− 0.0655	0.053	− 0.074	0.245	− 0.0553	0.102	− 0.128	**0.005**
WSS_diastole,avg_ (dynes/cm^2^)	1.66	** < 0.001**	1.59	**0.003**	0.927	**0.004**	1.55	**0.001**
WSS_diastole,peak_ (dynes/cm^2^)	0.297	** < 0.001**	0.299	** < 0.001**	0.283	**0.002**	0.423	**0.002**
OSI_avg_	575	**0.005**	268	0.245	244	0.066	308	**0.006**

Multivariable linear regression adjusting for patient sex, age at scan 1, and age at scan 2 was performed between each geometric and hemodynamic variable (with different vessel locations indicated by column) and the continuous outcome of RVSVi at Scan 2. Subjects with PVR between scans were excluded from this analysis. Effect estimates are interpreted as change in RVSVi (mL/m^2^) per unit increase in the baseline variable. Unadjusted p-values are provided. p-values that remained significant after Benjamini–Hochberg adjustment are bolded

*CI* confidence interval, *CMR* cardiovascular magnetic resonance, *LPA* left pulmonary artery, *MPA* main pulmonary artery, *OSI* oscillatory shear index, *OR* odds ratio, *RPA* right pulmonary artery, *taWSS* time-averaged wall shear stress, *WSS* wall shear stress.

**Table 6:** Multivariable linear regression results for RVEF at Scan 2

Variable	Whole vessel		MPA		LPA		RPA	
	Effect estimate	Unadjusted p-value	Effect estimate	Unadjusted p-value	Effect estimate	Unadjusted p-value	Effect estimate	Unadjusted p-value
Curvature* (cm^−1^)	–	–	–	–	− 2.13	0.791	2.82	0.811
Tortuosity*	–	–	–	–	− 18.3	0.081	17	0.513
WSS_steady,avg_ (dynes/cm^2^)	0.43	0.085	0.93	0.049	0.2	0.061	0.232	0.181
WSS_steady,peak_ (dynes/cm^2^)	0.0462	0.084	0.101	0.030	0.0476	0.070	0.0671	0.057
taWSS_avg_ (dynes/cm^2^)	0.527	0.061	0.841	0.075	0.226	0.058	0.214	0.223
taWSS_peak_ (dynes/cm^2^)	0.0535	0.065	0.103	0.016	0.0508	0.071	0.0747	0.056
WSS_systole,avg_ (dynes/cm^2^)	0.205	0.053	0.336	0.032	0.0742	0.048	0.0817	0.290
WSS_systole,peak_ (dynes/cm^2^)	0.018	0.085	0.0393	0.029	0.0183	0.072	0.0278	0.065
WSS_diastole,avg_ (dynes/cm^2^)	− 0.0964	0.598	− 0.116	0.546	− 0.0342	0.766	− 0.0461	0.798
WSS_diastole,peak_ (dynes/cm^2^)	− 0.00979	0.771	− 0.0114	0.739	− 0.00889	0.792	0.00626	0.901
OSI_avg_	− 64.8	0.367	− 75.1	0.284	− 2.62	0.952	− 14	0.722

Multivariable linear regression adjusting for patient sex, age at scan 1, and age at scan 2 was performed between each geometric and hemodynamic variable (with different vessel locations indicated by column) and the continuous outcome of RVEF at Scan 2. Subjects with PVR between scans were excluded from this analysis. Effect estimates are interpreted as change in RVEF (%) per unit increase in the baseline variable. Unadjusted p-values are provided. P-values that remained significant after Benjamini–Hochberg adjustment are bolded

*CI* confidence interval, *CMR* cardiovascular magnetic resonance, *LPA* left pulmonary artery, *MPA* main pulmonary artery, *OSI* oscillatory shear index, *OR* odds ratio, *RPA* right pulmonary artery, *taWSS* time-averaged wall shear stress, *WSS* wall shear stress.

#### Secondary Analysis: Geometric and CFD-Derived Variables in Association with PVR-Free Survival

In multivariable Cox proportional hazards models adjusting for patient sex and age at CMR (Table 7), we found that LPA WSS_steady,avg_ and RPA WSS_steady,peak_ were each significantly associated with time to PVR (hazard ratio [HR] 0.61 per unit increase, 95% confidence interval [CI] 0.39-0.96, p = 0.033 and HR 0.75, 95% CI 0.59-0.96, p = 0.020, respectively). Both of these results indicate that a higher WSS was associated with a decreased likelihood of PVR.

**Table 7:** Cox proportional hazards results for PVR-free survival

Variable	Whole vessel		MPA		LPA		RPA	
	HR (95% CI)	p–value	HR (95% CI)	p-value	HR (95% CI)	p-value	HR (95% CI)	p-value
Curvature* (cm^−1^)	–	–	–	–	0.9 (0.27–2.9)	0.854	0.6 (0.21–1.7)	0.326
Tortuosity*	–	–	–	–	0.57 (0.18–1.8)	0.342	1.9 (0.71–5.2)	0.202
WSS_steady,avg_ (dynes/cm^2^)	0.032 (0.00083–1.2)	0.063	0.069 (0.0036–1.3)	0.075	0.61 (0.39–0.96)	0.033	0.47 (0.21–1)	0.063
WSS_steady,peak_ (dynes/cm^2^)	0.93 (0.85–1)	0.086	0.89 (0.78–1)	0.066	0.93 (0.86–1)	0.101	0.75 (0.59–0.96)	0.02
taWSS_avg_ (dynes/cm^2^)	0.86 (0.62–1.2)	0.364	0.74 (0.5–1.1)	0.15	0.93 (0.79–1.1)	0.397	0.98 (0.87–1.1)	0.755
taWSS_peak_ (dynes/cm^2^)	0.99 (0.97–1)	0.722	0.98 (0.93–1)	0.428	0.99 (0.96–1)	0.665	0.96 (0.9–1)	0.251
WSS_systole,avg_ (dynes/cm^2^)	0.96 (0.87–1.1)	0.447	0.93 (0.82–1.1)	0.289	0.98 (0.93–1)	0.423	1 (0.95–1.1)	0.98
WSS_systole,peak_ (dynes/cm^2^)	1 (0.99–1)	0.714	1 (0.98–1)	0.607	1 (0.99–1)	0.655	0.99 (0.96–1)	0.278
WSS_diastole,avg_ (dynes/cm^2^)	1 (0.93–1.2)	0.422	1 (0.92–1.2)	0.544	1 (0.95–1.1)	0.498	1.1 (0.95–1.2)	0.328
WSS_diastole,peak_ (dynes/cm^2^)	1 (0.99–1)	0.49	1 (0.98–1)	0.702	1 (0.99–1)	0.403	1 (0.98–1.1)	0.311
OSI_avg_*	6.6 (1.3–33)	0.02	3.4 (1.2–9.7)	0.025	3 (0.88–10)	0.08	2.8 (0.66–12)	0.161

Cox proportional hazards analyses were performed between each geometric and hemodynamic variable and the outcome of pulmonic valve replacement (PVR), adjusting for patient sex and age at CMR. Variables with an asterisk (*) were normalized by the standard deviation of their distribution prior to performing Cox proportional hazards analysis in order to increase interpretability of the resulting hazard ratios. Bolded p-values are statistically significant (<0.05).

*CI* confidence interval, *CMR* cardiovascular magnetic resonance, *HR* hazard ratio, *LPA* left pulmonary artery, *MPA* main pulmonary artery, *OSI* oscillatory shear index, *RPA* right pulmonary artery, *taWSS* time-averaged wall shear stress, *WSS* wall shear stress.

From pulsatile simulations, we found that whole vessel OSI_avg_ (HR 6.6 per standard deviation increase, 95% CI 1.3 − 33, p = 0.020) and MPA OSI_avg_ (HR 3.4, 95% CI 1.17-9.7, p = 0.025) were both associated with time to PVR, indicating that a higher OSI_avg_ was associated with a higher likelihood of PVR in both of these vessel segments.

When applying ROC analysis for these variables to predict PVR, we found an optimal cutoff of 2.63 dynes/cm^2^ for LPA WSS_steady,avg_ and a cutoff of 22.4 dynes/cm^2^ for RPA WSS_steady,peak_. Additionally, we found a whole vessel OSI_avg_ cutoff of 0.22, and an MPA OSI_avg_ cutoff of 0.26. Kaplan Meier curves showing PVR-free survival for each of these cutoffs are shown in Figure 5.

## Discussion

Our work associates PA CFD-derived measurements with outcomes in patients with rToF. We demonstrate that several focal hemodynamic metrics in the main and branch PAs, including WSS_steady_, taWSS, WSS_systole_, WSS_diastole,_ and OSI are associated with RV measurements at follow-up scan, including RVEDVi, RVESVi, and RVSVi. We additionally showed that LPA WSS_steady,avg_, RPA WSS_steady,peak_, whole vessel OSI_avg_, and MPA OSI_avg_ are associated with time to PVR. We also provide a comprehensive cross-sectional description of geometric and hemodynamic metrics in rToF patient PAs that adds to the current body of literature in the largest study of CFD in the PAs of rToF patients to-date.

### Pulmonary Arterial Hemodynamics in Other Diseases

Historically, arterial shear stress has been studied in association with atherosclerosis in the systemic circulation; however, CFD and the role of WSS in the development and outcomes of pulmonary circulation-related diseases are currently areas of significant research effort. Among congenital heart diseases, much of the hemodynamic research has focused on the Fontan circulation in patients with Single Ventricle Disease (SVD). SVD patients who have a Fontan circulation have their superior and inferior vena cavae connected directly to their PAs, without the assistance of the right heart to bring blood to the lungs. Hemodynamic metrics in this circuit, such as power loss, have been shown to associate with outcomes and liver fibrosis in SVD patients with Fontan circulation [22–24]. Another study showed that Fontan patients have lower WSS compared to healthy controls [25]. Although the PA connections are different in SVD and rToF, analogies can be drawn between the relationships of the PAs with the upstream organ, the liver in SVD and the right heart in rToF.

Outside of congenital heart disease, PA hemodynamics have also been investigated extensively in pulmonary arterial hypertension (PAH), a disease characterized by high PA pressures and large, tortuous PAs. Studies have demonstrated an association of higher OSI with PAH in both adult and pediatric patients [26, 27], and additionally, recent *in vitro* work has shown that high WSS in the distal PAs can induce endothelial to mesenchymal transition, leading to the development of PAH [28]. Among other diseases, PA OSI has been shown to be higher in patients with systemic sclerosis [29], and a CFD study showed that patients with chronic thromboembolic pulmonary hypertension had decreased WSS and increased OSI compared to controls [30]. Altogether, these studies suggest important, potentially mechanistic links between PA WSS, OSI, and the development of PA disease.

### Pulmonary Arterial Geometry and Hemodynamics in rToF

There has been limited investigation into the relationship between geometry and longitudinal RV remodeling in rToF. One study suggested that elevated intracardiac RV wall shear stress contributed to RV wall thickening [31], and another study demonstrated that larger PA bifurcation angle was associated with risk of reoperation [9]. Louvelle et al. performed a cross-sectional analysis of 16 pediatric rToF patients and found that more tortuous anatomies in both the right and left PAs were associated with higher energy losses [8]. They posited that these higher energy losses may be related to future risk of RV overload. Boumpouli et al. performed a similar cross-sectional analysis in 7 rToF patients ranging from 5 to 54 years old [13]. They found higher curvature and tortuosity in the LPA compared to the RPA, and noted that the LPA was often a site of complex flow. Recently, this same group compared an rToF cohort to a group of healthy volunteers and showed that rToF patients had higher taWSS [32]. These studies emphasize the importance of using patient-specific models and boundary conditions, but have not yet linked geometry and hemodynamics with RV remodeling-related outcomes. It is important to also note that hemodynamics themselves represent a combination of vessel structure (inclusive of features such as stenosis and curvature) as well as cardiac function (which influences flow).

In order to investigate our hypothesis that PA geometry and hemodynamics are associated with RV remodeling, we studied 22 patients, ranging from 1 to 51 years old at first CMR, who received MRA imaging in systole at baseline and also had a follow-up scan at which cardiac function was assessed. In a cross-sectional analysis of our cohort at their baseline scan, we saw that LPA curvature was greater than RPA curvature (similar to Louvelle et al.), and that for WSS_steady,avg_, taWSS_average_, and WSS_systole,avg_, LPA WSS was greater than RPA WSS, which was greater than MPA WSS. We hypothesize that these differences are mainly due to the differences in size between the LPA, RPA, and MPA as the MPA bifurcates. Additionally, variations in the PAs are common in ToF, occurring in almost 20% of patients, with LPA stenosis being the most common abnormality, occurring 10% of the time [33]. Differences may also be attributed to variations in the angles between the LPA, RPA, and MPA. We also saw higher OSI_avg_ in the MPA, which was greater than both LPA OSI_avg_ and RPA OSI_avg_. This is most likely due to the proximity of the MPA to the inlet, meaning that it is more directly exposed to changes in inflow from PR. A high OSI_avg_ was expected in our cohort due to significant PR (a median of 40%) altering the direction of flow during the cardiac cycle.

In PVR-free survival analyses, we found that LPA WSS_steady,avg_ and RPA WSS_steady,peak_ were associated with a decreased likelihood of PVR. From our pulsatile results, we found that whole vessel OSI_avg_ and MPA OSI_avg_ were each associated with an increased likelihood of PVR. These findings suggest that branch WSS calculated via steady methods and whole vessel and MPA OSI from pulsatile studies may be important factors to consider in beginning to understand the mechanisms underlying the need for PVR in rToF patients. We hypothesize that these findings may be related to regurgitant flow back through the pulmonary valve (i.e. more regurgitation leads to higher OSI). The significance of CFD-derived hemodynamic, and not geometric, variables in this analysis points to the value of CFD to characterize and understand blood flow in rToF PAs.

We also studied our geometric and CFD-derived measurements in association with RV remodeling metrics derived from RV size and function in follow-up CMR studies, finding several significant associations between vessel-specific hemodynamics and RV measurements. These relationships generally showed that, as WSS_steady_, taWSS, and WSS_systole_ increase, RVEDVi, RVESVi, and RVSVi, decrease, while WSS_diastole_ showed a positive relationship with these metrics. These findings may be reflective of the degree of regurgitant flow in these patients, but further study will be important to elucidate the mechanisms of these relationships.

### Limitations

There are several limitations to our work, the main being our sample size of 22 subjects which likely meant we were underpowered to detect all of the true differences from our analyses. Our cohort also consisted of a wide range of ages, which can affect hemodynamic measurements; however, we chose to adjust all of our models for age to mitigate this effect. Another limitation is that not all patients with rToF are referred for CMR, meaning our patients may be expected to have been sicker than the rToF population as a whole. As the speed and utility of CMR continue to increase, we expect its utilization for the monitoring of rToF to increase as well, leading to increased generalizability of studies performed using rToF CMR.

Because of our small sample size, we also had a limited number of events for the PVR-free Cox proportional hazards analysis. While it has been shown that confidence interval coverage and bias are acceptable with less than 10 events per variable [34], it remains highly likely that this analysis was underpowered. However, the fact that we did find significant results at this low power motivates further studies in this field.

Time-resolved simulation parameters were used for the steady-state computations, leading to a computationally inefficient setup. Although convergence was achieved well before the full 25,000 steps, we retained these settings to ensure consistency across all simulation cases and to facilitate comparison between steady and pulsatile flow fields. Future work could optimize these parameters to reduce computational cost without compromising accuracy.

One assumption made in our models was the rigid wall assumption, which vastly simplifies the computational cost of the simulations. To maximize the accuracy of our simulations, we used images acquired in systole so that any stenoses would not be exaggerated by diastolic backwards flow making the vessel smaller. Similar methods were used by both Louvelle et al. and Boumpouli et al. in their studies of PA CFD in rToF [8, 13]. We constructed our geometries and performed our simulations using methods similar to both of these studies to enable reasonable comparisons between their findings and our own. Although a previous comparison between rigid wall and fluid-structure interaction (FSI) models in the pulmonary artery demonstrated that the rigid wall assumption overestimates WSS [35], it is unclear if these findings would translate to our results, as our work used a systolic geometry in comparison to the authors’ diastolic geometry, and our patients experienced significant pulmonary regurgitation, which did not occur in the other study.

Additionally, we opted to use a simple resistance boundary condition. This approach enabled the isolation of upstream geometric and flow-related factors without the confounding influence of additional calibration parameters. While more physiologically detailed boundary conditions such as Resistor-Capacitor-Resistor (RCR) models can better capture vascular impedance, particularly in the pulmonary circulation, their application requires distal pressure or flow data that were not available in our study cohort.

### Future Directions

This work requires significant further exploration and validation before it may affect patient care. Our results suggest that standardized use of MRA among rToF patients may be warranted to fully characterize patients’ likelihood of PVR as well as RV remodeling after initial repair. As an alternative to MRA, which uses a gadolinium-based contrast, we could consider using alternative non-contrast imaging sequences, such as the axial HASTE (Half-Fourier Acquisition Single-shot Turbo spin Echo) sequence that is typically acquired for all patients, to perform our segmentations. We also believe it would be valuable to study PA remodeling over time to assess whether geometry and hemodynamics in the PAs change over time.

4D flow data would have enabled us to calculate WSS directly from CMR; however, 4D flow is not yet regularly collected at our institution. Additionally, 4D flow data is typically lower resolution compared to MRA. As the usage of CMR increases and as protocols begin to change, we may begin to have this data for our rToF patients. Finally, as the PA is a highly dynamic vessel, we believe it will be important to perform future studies using FSI models that account for the movement of the vessel between systole and diastole and the subsequent effects on hemodynamic measurements.

## Conclusion

In this study, we provided geometric and hemodynamic characterizations of the PAs in a cohort of rToF patients with longitudinal follow-up data. We demonstrated novel associations of WSS and OSI with PVR, and WSS and OSI with follow-up RV metrics. Further study is warranted to (1) validate these findings in larger cohorts, and (2) elucidate the mechanisms by which WSS and OSI are related to PVR and RV remodeling.

## Citation Diversity Statement

Recent work in several fields of science has identified a bias in citation practices such that papers from women and other minority scholars are under-cited relative to the number of such papers in the field [36–44]. Here we sought to proactively consider choosing references that reflect the diversity of the field in thought, form of contribution, gender, race, ethnicity, and other factors. First, we obtained the predicted gender of the first and last author of each reference by using databases that store the probability of a first name being carried by a woman [40, 45]. By this measure and excluding self-citations to the first and last authors of our current paper), our references contain 9.26% woman(first)/woman(last), 21.48% man/woman, 15.74% woman/man, and 53.53% man/man. This method is limited in that a) names, pronouns, and social media profiles used to construct the databases may not, in every case, be indicative of gender identity and b) it cannot account for intersex, non-binary, or transgender people. Second, we obtained predicted racial/ethnic category of the first and last author of each reference by databases that store the probability of a first and last name being carried by an author of color [46, 47]. By this measure (and excluding self-citations), our references contain 10.81% author of color (first)/author of color(last), 12.51% white author/author of color, 19.13% author of color/white author, and 57.54% white author/white author. This method is limited in that a) names and Florida Voter Data to make the predictions may not be indicative of racial/ethnic identity, and b) it cannot account for Indigenous and mixed-race authors, or those who may face differential biases due to the ambiguous racialization or ethnicization of their names. We look forward to future work that could help us to better understand how to support equitable practices in science.

## Supplementary Information

Below is the link to the electronic supplementary material.

Electronic supplementary material 1 (PDF 1244 kb)
