# Supplementary material for: Pulmonary Artery Shear Stress and Oscillatory Shear Index are Associated with Right Ventricular Remodeling in Repaired Tetralogy of Fallot
Source: Ann Biomed Eng. 2025 Jul 3;53(9):2206–22. doi: 10.1007/s10439-025-03793-0 (PMC12391157; doi:10.1007/s10439-025-03793-0)
Supplement: Supplementary file 1 — Electronic supplementary material 1 (PDF 1244 kb) [file 10439_2025_3793_MOESM1_ESM.pdf]

Supplementary Information

Title: Pulmonary Artery Shear Stress and Oscillatory Shear Index are Associated with Pulmonary Valve Replacement and Right Ventricular Remodeling in Repaired Tetralogy of Fallot

Journal: Annals of Biomedical Engineering

Authors: Elizabeth W. Thompson†, Anindro Bhattacharya, Fengling Hu, Taki Shinohara, Paris Perdikaris, Kevin K. Whitehead, Elizabeth Goldmuntz, Mark A. Fogel, Walter R. Witschey

†Corresponding author:
Elizabeth W. Thompson
Perelman School of Medicine, University of Pennsylvania, Philadelphia, PA, United States
elizabeth.thompson@pennmedicine.upenn.edu

Formatted: Highlight

Supplementary Figures

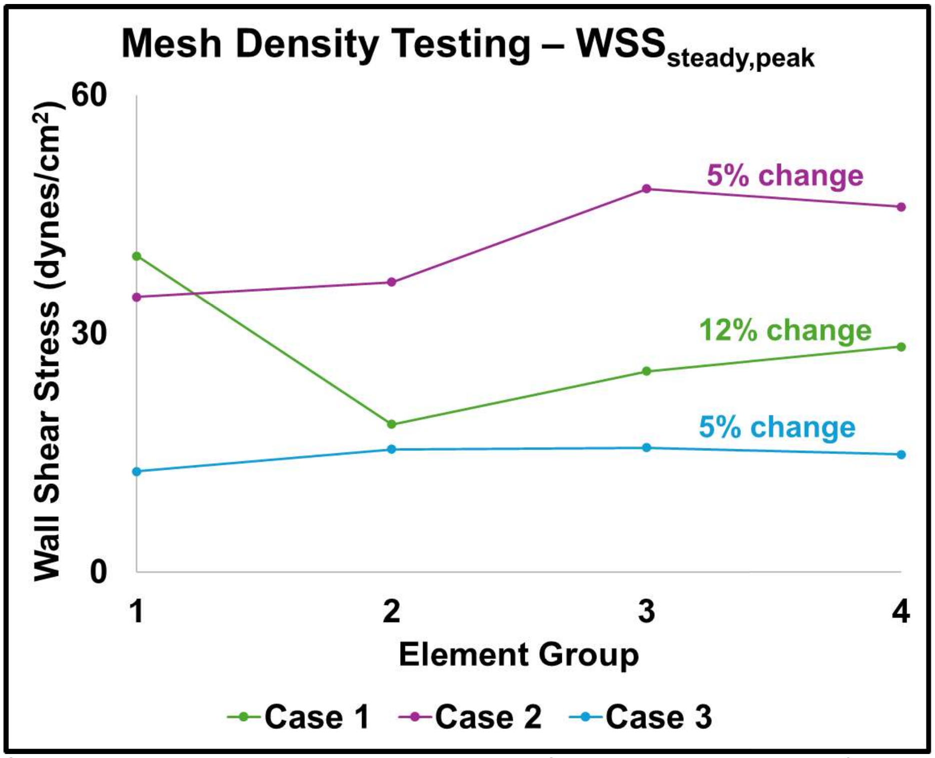

Supplemental Fig. 1 Mesh density testing. We performed mesh density testing for three cases to determine the optimal number of elements for each mesh. The element groups were as follows (all numbers approximate): 1) 1 million elements, 2) 2.5 million elements (similar to Boumpouli et al.(1)), 3) 5 million elements, and 4) 8 million elements (similar to Louvelle et al. (2)). Our variable of interest was peak wall shear stress ($WSS_{steady,peak}$) across the whole vessel, and we *a priori* set a goal to see stability of this variable measured as a change of <15% between element group simulations. The percent change in $WSS_{steady,peak}$ is shown on the graph between the last two groups (all values <15%), signifying that the results stabilized

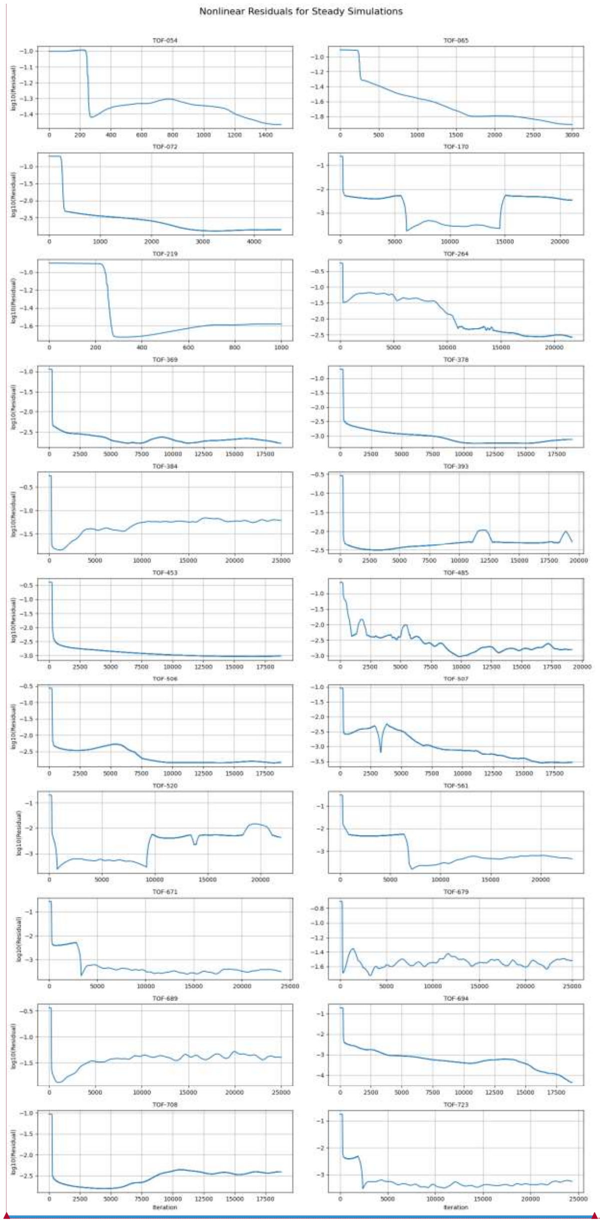

Supplemental Fig. 2 Log non-linear residuals over iterations for all steady-state simulations. 2.5 seconds of flow at a time step of 0.0001 seconds was simulated for all cases.

Formatted: Highlight

Formatted: Highlight

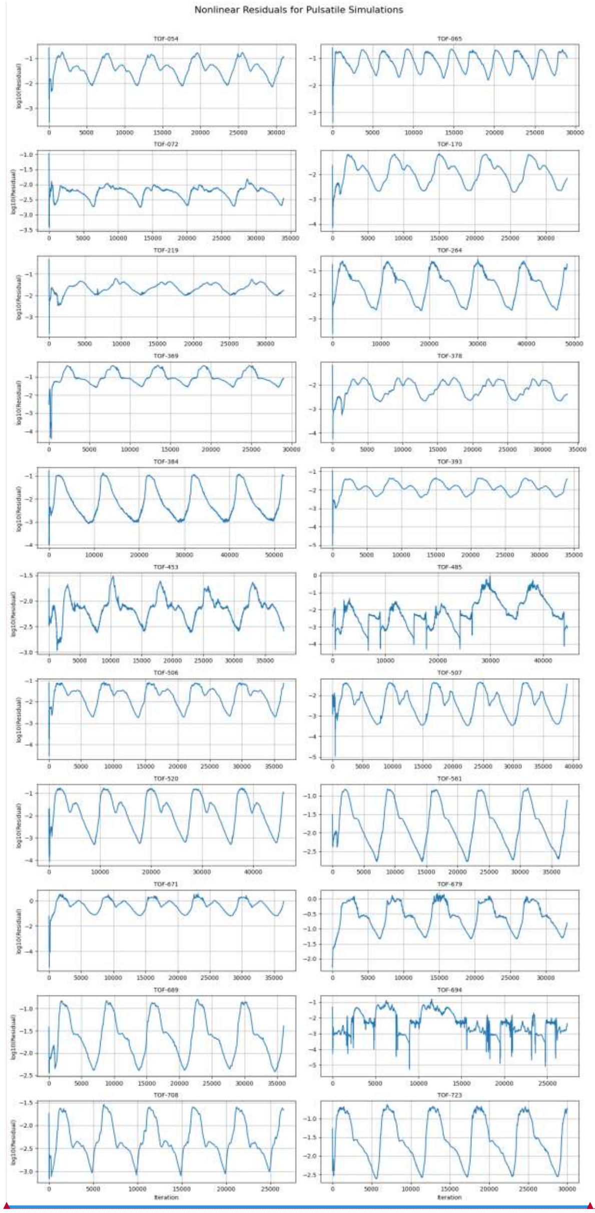

Supplemental Fig. 3 Log non-linear residuals over iterations for all pulsatile-state simulations. Five cardiac cycles were simulated based on the patient's heart rate and a time step of 0.0001 seconds.

Formatted: Highlight

Formatted: Highlight

Commented [BT1]: Reviewer 2: Comment 2b; Reviewer 3 Comment 3 (convergence supplementary figures)

Formatted: Highlight

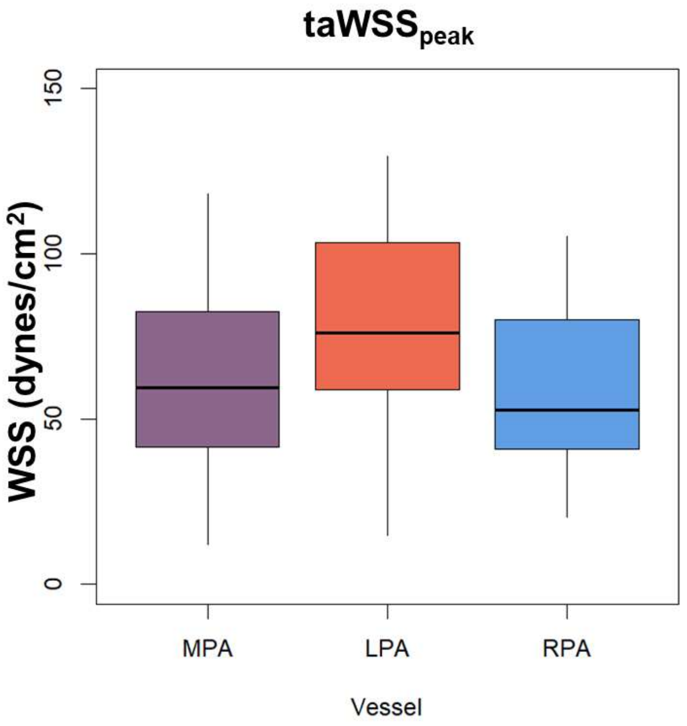

Supplemental Fig. 42 Pulsatile CFD Peak taWSS comparisons between the main, left, and right pulmonary arteries in patients with rToF. Vessel-specific peak time-averaged wall shear stress (taWSS) was extracted from pulsatile CFD simulations performed on pulmonary artery models from patients with rToF. There were no significant differences between peak taWSS in the MPA (purple), the LPA (red), and the RPA (blue). *CFD = computational fluid dynamics; ;LPA = left pulmonary artery; MPA = main pulmonary artery; RPA = right pulmonary artery; rToF = repaired tetralogy of Fallot*

Formatted: Highlight

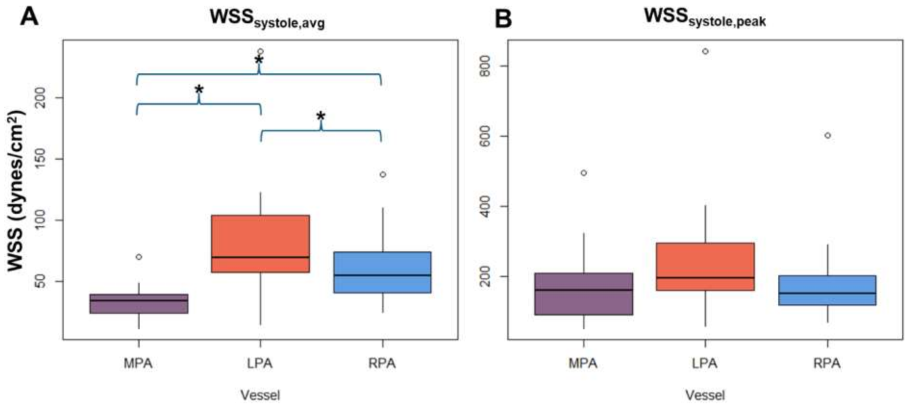

Supplemental Fig. 5~~3~~ Pulsatile CFD systolic WSS comparisons between the main, left, and right pulmonary arteries in patients with rToF. Vessel-specific wall shear stress (WSS) was extracted from pulsatile CFD simulations performed on pulmonary artery models from patients with rToF at the peak systolic flow timepoint. Among average systolic WSS measurements, the LPA (red) had greater WSS than both the RPA (blue) and MPA (purple), and RPA WSS was greater than MPA WSS (**A**). Peak systolic WSS did not differ significantly between vessels (**B**). *CFD = computational fluid dynamics; LPA = left pulmonary artery; MPA = main pulmonary artery; RPA = right pulmonary artery; rToF = repaired tetralogy of Fallot.* * denotes $p<0.05$

Formatted: Highlight

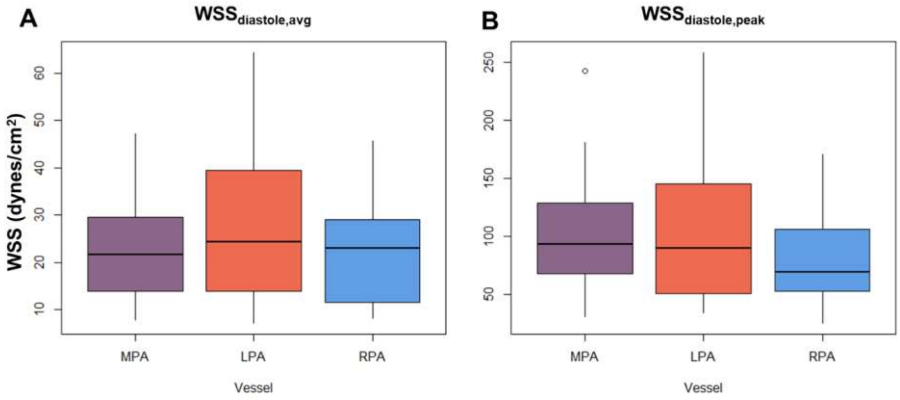

Supplemental Fig. 64 Pulsatile CFD diastolic WSS comparisons between the main, left, and right pulmonary arteries in patients with rToF. Vessel-specific wall shear stress (WSS) was extracted from pulsatile CFD simulations performed on pulmonary artery models from patients with rToF at the peak diastolic flow timepoint (most negative flow). Neither average diastolic WSS (**A**) nor peak diastolic WSS (**B**) showed significant differences between vessel segments. *CFD = computational fluid dynamics; LPA = left pulmonary artery; MPA = main pulmonary artery; RPA = right pulmonary artery; rToF = repaired tetralogy of Fallot*

Formatted: Highlight

Supplementary Tables

Supplemental Table 1: Change in non-linear residuals between last 100 iterations in steady case simulations.

Patient	Absolute Difference in Non-Linear Residual	Percent Difference in Non-Linear Residual	Absolute Difference in Log Non-Linear Residual (%)	Percent Difference in Log Non-Linear Residual (%)
054	1.01E-06	0.043148891	0.000187353	0.007117264
065	6.02E-06	0.900492135	0.003928502	0.123740833
072	5.65E-07	0.039910729	0.000173365	0.006085478
170	2.55E-06	0.073829102	0.000320754	0.013034137
219	3.81E-06	0.311646308	0.001355576	0.046535955
264	1.27E-05	0.472107884	0.002055194	0.079994189
369	5.55E-06	0.332041182	0.001444436	0.052022158
378	1.79E-06	0.236944369	0.001027819	0.032915751
384	5.09E-06	0.302898972	0.00131747	0.047480656
393	0.000936461	20.68787825	0.081663652	3.483607675
453	1.17E-06	0.121320816	0.00052657	0.017467547
485	1.74E-05	1.111356832	0.004799938	0.171120438
506	1.31E-05	0.887168638	0.003835934	0.135432217
507	5.54E-07	0.188758731	0.000818996	0.023185779
520	2.13E-05	0.492540291	0.002144361	0.090704698
561	7.78E-05	0.342619888	0.001490534	0.090674307
671	6.64E-05	0.502110407	0.002186131	0.116360179
679	1.64E-05	1.673943973	0.00720967	0.239545046
689	4.27E-06	0.409290021	0.001781172	0.059738717
694	1.84E-06	4.040366849	0.017911421	0.412583804
708	2.74E-06	0.069720457	0.000302898	0.012595204

Supplemental Table 2: Comparison of non-linear residuals at systole and diastole over the last two cardiac cycles in pulsatile simulations.

Patient	Absolute Difference in Non-Linear Residual		Percent Difference in Non-Linear Residual (%)		Absolute Difference in Log Non-Linear Residual		Percent Difference in Log Non-Linear Residual	
	Systole	Diastole	Systole	Diastole	Systole	Diastole	Systole	Diastole
054	0.001167281	0.00024015	3.986273767	3.058739477	0.016665277	0.013492681	1.086828164	0.640954025
065	0.004812969	0.001219188	18.66739866	5.40602676	0.094925776	0.022661534	5.9798995	1.37614488
072	0.001022163	0.000137766	16.57661426	7.986461941	0.072166153	0.032472157	3.268476397	1.175166909
170	0.000908969	5.38E-05	6.342222836	2.610933339	0.026515813	0.011159244	1.438113674	0.415469028
219	0.003445781	0.000792578	12.15805125	7.930265923	0.049222192	0.035895168	3.182552768	1.794744225
264	0.0029285	0.000190609	9.912860092	8.497261877	0.040109607	0.036389508	2.622136477	1.373871222
369	0.002374531	0.002388281	3.192451866	9.580202097	0.014106725	0.039150146	1.250007904	2.442663027
378	0.000455184	0.000120784	5.074881817	5.766260364	0.021465294	0.024340641	1.048455435	0.908577077
384	0.001126444	5.50E-05	16.6812353	6.626579655	0.083335709	0.027306142	3.840509871	0.886215716
393	0.001077656	0.000383291	9.895493596	9.298240407	0.042930629	0.041324701	2.187888024	1.733285995
453	0.000462581	0.000175781	7.613961614	7.546116589	0.031830041	0.033125984	1.436216465	1.258405821
485	0.0016325	8.52E-05	7.091245058	3.059763535	0.030771133	0.013286312	1.879207951	0.519983822
506	0.000761688	8.78E-05	3.910752998	4.750612072	0.01712897	0.020638803	1.001491189	0.755195983
507	0.000312347	2.20E-05	9.311478408	5.990641853	0.041606741	0.027035761	1.681827392	0.787211013
520	0.00063225	6.32E-05	4.534150204	11.99326675	0.020140294	0.049173162	1.08546736	1.4996029
561	0.00168575	7.19E-05	8.440654137	4.352634359	0.039551611	0.018780514	2.327472202	0.675146056
671	0.005431562	0.004062344	1.811462629	5.612986225	0.007942541	0.025161911	1.518350362	2.206985218
679	0.002866563	0.004119312	1.286455139	8.593788113	0.005527351	0.036936901	0.847708983	2.801090375
689	0.000962313	1.62E-05	3.69630053	0.401368043	0.015763256	0.0017467	0.994851664	0.072947916
694	0.000765972	5.74E-05	5.807456012	1.335097966	0.025207305	0.005798085	1.341166764	0.245021751
708	0.000106181	4.60E-05	3.283307984	5.120210733	0.013992523	0.022826732	0.561895272	0.749258976
723	0.001006281	0.000131803	4.983068449	5.543057607	0.022558995	0.022933967	1.331103668	0.87407613

Commented [BT2]: Reviewer 2: Comment 2b;

References

1. Boumpouli M, Sauvage EL, Capelli C, Schievano S, Kazakidi A. Characterization of Flow Dynamics in the Pulmonary Bifurcation of Patients With Repaired Tetralogy of Fallot: A Computational Approach. Front Cardiovasc Med 2021;8:703717.
2. Louvelle L, Doyle M, Van Arsdell G, Amon C. The Effect of Geometric and Hemodynamic Parameters on Blood Flow Efficiency in Repaired Tetralogy of Fallot Patients. Ann Biomed Eng 2021;49:2297-2310.
